# Determination of Reactivity Ratios from Binary Copolymerization Using the k-Nearest Neighbor Non-Parametric Regression

**DOI:** 10.3390/polym13213811

**Published:** 2021-11-04

**Authors:** Iosif Sorin Fazakas-Anca, Arina Modrea, Sorin Vlase

**Affiliations:** 1 AGIMED Sovata, 545500 Sovata, Romania; d-mec@unitbv.ro; 2 Pharmacy, Science and Technology George Emil Palade Targu Mures, University of Medicine, 300134 Targu Mures, Romania; 3 Department of Mechanical Engineering, Transilvania University of Brasov, B-dul Eroilor 20, 500036 Brasov, Romania; 4 Romanian Academy of Technical Sciences, B-dul Dacia 26, 030167 Bucharest, Romania

**Keywords:** k-NN regression, reactivity ratios, optimization, copolymerization, error estimation, propagation rate, monomers

## Abstract

This paper proposes a new method for calculating the monomer reactivity ratios for binary copolymerization based on the terminal model. The original optimization method involves a numerical integration algorithm and an optimization algorithm based on k-nearest neighbour non-parametric regression. The calculation method has been tested on simulated and experimental data sets, at low (<10%), medium (10–35%) and high conversions (>40%), yielding reactivity ratios in a good agreement with the usual methods such as intersection, Fineman–Ross, reverse Fineman–Ross, Kelen–Tüdös, extended Kelen–Tüdös and the error in variable method. The experimental data sets used in this comparative analysis are copolymerization of 2-(*N*-phthalimido) ethyl acrylate with 1-vinyl-2-pyrolidone for low conversion, copolymerization of isoprene with glycidyl methacrylate for medium conversion and copolymerization of *N*-isopropylacrylamide with *N*,*N*-dimethylacrylamide for high conversion. Also, the possibility to estimate experimental errors from a single experimental data set formed by n experimental data is shown.

## 1. Introduction

Technological development brings with it the need to create new polymers with predefined physico-chemical properties. It is well known that the physico-chemical properties of polymers are given by their microstructure, and the microstructure is determined by the reaction kinetics. By the nature of the monomers used in the copolymerization reaction and by a controlled kinetics, specific microstructures can be obtained such as: polymers with amorphous or crystalline areas, polymers with large molecular masses, branching polymers, crosslinked polymers or more other microstructure types. All these microstructure types have great influence on the mechanical and chemical behavior of the resulting polymers. The possibilities to obtain any kind of mechanical or chemical properties of copolymers are practically unlimited, but there exists only one limitation to our imagination. The mechanism of binary copolymerization in which it is considered that only the last structural unit attached to the polymer chain influences the growth mode of the polymer is described by the following kinetic relations [1]:

$$P_{n}-M_{1}^{*}+M_{1}\overset{k_{11}}{\to }P_{n+1}-M_{1}^{*}$$

$$P_{n}-M_{1}^{*}+M_{2}\overset{k_{12}}{\to }P_{n+1}-M_{2}^{*}$$

$$P_{n}-M_{2}^{*}+M_{1}\overset{k_{21}}{\to }P_{n+1}-M_{1}^{*}$$

$$P_{n}-M_{2}^{*}+M_{2}\overset{k_{22}}{\to }P_{n+1}-M_{2}^{*}$$

where *P_n_*—growing polymer chain, *M*_1_^*^, *M*_2_^*^—the active center on monomers, $k_{11}$, $k_{12}$, $k_{21}$, $k_{22}$—propagation rate constants.

The transformation of the above kinetic equations into a mathematical model that connects the kinetic evolution, and the microstructure of the formed copolymer is obtained using the mathematical equations of a first order kinetics, described by the following equations:

(1) $$-\frac{dM_{1}}{dt}=k_{11}\left[M_{1}^{*}\right]\left[M_{1}\right]+k_{21}\left[M_{2}^{*}\right]\left[M_{1}\right]$$

(2) $$-\frac{dM_{2}}{dt}=k_{22}\left[M_{2}^{*}\right]\left[M_{2}\right]+k_{12}\left[M_{1}^{*}\right]\left[M_{2}\right]$$

where −*dM*_1_/*dt*, −*dM*_2_/*dt*—rate of monomers consumption, [*M*_1_], [*M*_2_]—molar concentration of monomers in feed, [*M*_1_^*^], [*M*_2_^*^]—molar concentration of polymer chain growth active centers.

It is obvious that both the reaction mechanism and the kinetic Equations (1) and (2) are not entirely correct because they do not consider the initiation reaction, the termination reaction, and the transfer reaction of the active center. However, to be able to generate a mathematical model in which parameters that cannot be measured do not appear, it is mandatory to impose the stationary state condition described by relation (3):

(3) $$k_{12}\left[M_{1}^{*}\right]\left[M_{2}\right]=k_{21}\left[M_{2}^{*}\right]\left[M_{1}\right]$$

Considering the above, several authors [2,3,4,5] have proposed various mathematical solutions that describe the connection between the microstructure of the copolymer and the kinetics of the reaction. Thus, Alfrey Jr. and Goldfinger [2] propose the following relation (4):

(4) $$\frac{d\left[M_{2}\right]}{d\left[M_{1}\right]}\approx \frac{m_{2}}{m_{1}}=\frac{\left[M_{2}\right]}{\left[M_{1}\right]}\cdot r_{1}\frac{\frac{1}{r_{2}}\left[M_{2}\right]+\left[M_{1}\right]}{r_{1}\left[M_{2}\right]+\left[M_{1}\right]}$$

Mayo and Lewis [3] propose relation (5):

(5) $$\frac{d\left[M_{1}\right]}{d\left[M_{2}\right]}\approx \frac{m_{1}}{m_{2}}=\frac{\left[M_{1}\right]}{\left[M_{2}\right]}\cdot \frac{r_{1}\left[M_{1}\right]+\left[M_{2}\right]}{\left[M_{1}\right]+r_{2}\left[M_{2}\right]}$$

and Wall [4] and Skeist [5] propose the following form:

(6) $$\frac{d\left[M_{1}\right]}{d\left[M_{1}\right]+d\left[M_{2}\right]}\approx m_{1}=\frac{r_{1}\left[M_{1}^{2}\right]+\left[M_{1}\right]\left[M_{2}\right]}{r_{1}\left[M_{1}^{2}\right]+2\left[M_{1}\right]\left[M_{2}\right]+r_{2}\left[M_{2}^{2}\right]}$$

where,

(7) $$r_{1}=\frac{k_{12}}{k_{11}}\;\;\;\;\mathrm{and}\;r_{2}=\frac{k_{21}}{k_{22}}$$

*r*_1_, *r*_2_—reactivity ratios of monomers.

After all, it is easy to see that the Equations (4)–(6) are nested equations, and the most common form is that described by Equation (5). This mathematical model is a differential one that makes the connection between the reaction kinetics and the instantaneous composition of the copolymer and can be used for experimental data which have conversion below 10%.

For experimental data with conversions greater than 10% it is necessary to use the integral form of the differential equation, the equation makes the connection between the reaction kinetics and the global composition of the copolymer. The integral equation proposed by Mayo and Lewis [3] has the form:

(8) $$\log\frac{\left[M_{2}\right]}{\left[M_{2}^{0}\right]}=\frac{r_{2}}{1-r_{2}}\cdot \log\frac{\left[M_{1}\right]\cdot \left[M_{2}^{0}\right]}{\left[M_{2}\right]\cdot \left[M_{1}^{0}\right]}-\frac{1-r_{1}r_{2}}{\left(1-r_{2}\right)\left(1-r_{1}\right)}\cdot \log\frac{\left(r_{1}-1\right)\frac{\left[M_{1}\right]}{\left[M_{2}\right]}-\left(r_{2}+1\right)}{\left(r_{1}-1\right)\frac{\left[M_{1}^{0}\right]}{\left[M_{2}^{0}\right]}-\left(r_{2}+1\right)}\;\;,$$

where [*M*_1_^0^], [*M*_2_^0^]—initial molar concentration of monomers in feed, [*M*_1_], [*M*_2_]—molar concentration of monomers in feed at given conversion.

Integrating the equation proposed by Wall [4] and Skeist [5], Meyer and Lowry [6] obtain the following mathematical solution:

(9) $$\frac{\left[M_{1}\right]+\left[M_{2}\right]}{\left[M_{2}^{0}\right]+\left[M_{2}^{0}\right]}=\frac{M}{M^{0}}=X={\left(\frac{f_{1}}{f_{1}^{0}}\right)}^{\alpha }{\left(\frac{f_{2}}{f_{2}^{0}}\right)}^{\beta }{\left(\frac{f_{1}^{0}-\delta }{f_{1}-\delta }\right)}^{\gamma }$$

where

(10) $$\;\;\;f_{1}=\frac{\left[M_{1}\right]}{\left[M_{1}\right]+\left[M_{2}\right]}=1-f_{2};\;\alpha =\frac{r_{2}}{1-r_{2}};\;\;\beta =\frac{r_{1}}{1-r_{1}};\;\;\gamma =\frac{1-r_{1}r_{2}}{\left(1-r_{1}\right)\left(1-r_{2}\right)};\;\;\delta =\frac{1-r_{2}}{2-r_{1}-r_{2}},$$

*X*—conversion.

As can be seen, Equations (8) and (9) also are nested equations.

Into a *r*_1_, *r*_2_ coordinate system, the Equations (3) and (9) proposed by Mayo and Lewis [3] describe a line for each experimental point of an experimental data set. Taking account of this Equation (5) can be rewritten as:

(11) $$r_{2}={\left(\frac{M_{1}}{M_{2}}\right)}^{2}\cdot \frac{m_{2}}{m_{1}}\cdot r_{1}+\frac{M_{1}}{M_{2}}\cdot \left(\frac{m_{2}}{m_{1}}-1\right)$$

and Equation (9) has the following form:

(12) $$r_{2}=\frac{\log\frac{\left[M_{2}^{0}\right]}{\left[M_{2}\right]}-\frac{1}{p}\log\frac{1-p\frac{\left[M_{1}\right]}{\left[M_{2}\right]}}{1-p\frac{\left[M_{1}^{0}\right]}{\left[M_{2}^{0}\right]}}}{\log\frac{\left[M_{1}^{0}\right]}{\left[M_{1}\right]}+\log\frac{1-p\frac{\left[M_{1}\right]}{\left[M_{2}\right]}}{1-p\frac{\left[M_{1}^{0}\right]}{\left[M_{2}^{0}\right]}}}$$

where

(13) $$p=\frac{1-r_{1}}{1-r_{2}}$$

By intersecting two lines thus obtained, are obtained the reactivity ratios as a solution that satisfy the parameters of the two experimental points considered. If we have n experimental points, we obtain m solutions of the experimental data set. The number of solutions m of an experimental data set is obtained with the relation:

(14) $$m=C_{n}^{2}=\frac{n\left(n-1\right)}{2}$$

Unfortunately, Mayo and Lewis [3] in their paper do not offer a solution for finding the best solution of reactivity ratios for the situation where *n* > 2. Since the publication of the intersection method [3] a few authors [7,8,9,10] have proposed various solutions to find the best value of the reactivity ratios from (2, m) matrix of solutions. An interesting solution for finding the best pair of values r_1_, r_2_ from the matrix of solutions obtained by the intersection method is proposed by Abdollahi et al. [10] (ANA). These authors consider that the optimal values of reactivity ratios *r*_1_^0^, *r*_2_^0^ are that which has the smallest distance from all calculated lines using the Equation (5) for all experimental points. To determine the optimal values *r*_1_^0^, *r*_2_^0^ the authors rewrite Equation (5) in the following form:

(15) $$r_{1}\left[M_{1}^{2}\right]\left(m_{1}-1\right)+r_{2}\left[M_{2}^{2}\right]+\left[M_{1}\right]\left[M_{2}\right]\left(2m_{1}-1\right)=0$$

The sum of the squares of the distance from the optimal point *r*_1_^0^, *r*_2_^0^ at each line is calculated with the relation:

(16) $$\sum d_{i}^{2}=\frac{{\langle \{r_{1}^{o}\left[M_{1}^{2}\right]\left(m_{1}-1\right)+r_{2}^{o}\left[M_{2}^{2}\right]+\left[M_{1}\right]\left[M_{2}\right]\left(2m_{1}-1\right)\}}_{i}\rangle^{2}}{{\left\{r_{1}^{o}\left[M_{1}^{2}\right]\left(m_{1}-1\right)\right\}}_{i}^{2}+{\left[M_{2}^{2}\right]}_{i}^{2}}=f\left(r_{1}^{o},r_{2}^{o}\right)$$

where *i*—denote the number of the experimental point from experimental data set.

To calculate the minimum distance from the optimal point *r*_1_^0^, *r*_2_^0^ to each line, the partial derivatives to *r*_1_^0^ and *r*_2_^0^ of the function *f*(*r*_1_^0^, *r*_2_^0^) respectively, are both of them set to zero. The partial derivatives equations are described by:

(17) $$\frac{\partial f}{\partial r_{1}^{o}}=r_{1}^{o}\sum \frac{2{\left[r_{1}^{o}M_{1}^{2}\left(m_{1}-1\right)\right]}_{i}^{2}}{{\left[r_{1}^{o}M_{1}^{2}\left(m_{1}-1\right)\right]}_{i}^{2}+{\left[M_{2}^{2}\right]}_{i}^{2}}+r_{2}^{0}\sum \frac{2{\left[r_{1}^{o}M_{1}^{2}\left(m_{1}-1\right)\right]}_{i}{\left[M_{2}^{2}\right]}_{i}}{{\left[r_{1}^{o}M_{1}^{2}\left(m_{1}-1\right)\right]}_{i}^{2}+{\left[M_{2}^{2}\right]}_{i}^{2}}+\sum \frac{2{\left[r_{1}^{o}M_{1}^{2}\left(m_{1}-1\right)\right]}_{i}{\left[M_{1}M_{2}\left(2m_{1}-1\right)\right]}_{i}}{{\left[r_{1}^{o}M_{1}^{2}\left(m_{1}-1\right)\right]}_{i}^{2}+{\left[M_{2}^{2}\right]}_{i}^{2}}=0$$

(18) $$\frac{\partial f}{\partial r_{2}^{o}}=r_{1}^{o}\sum \frac{2{\left[r_{1}^{o}M_{1}^{2}\left(m_{1}-1\right)\right]}_{i}{\left[M_{2}^{2}\right]}_{i}}{{\left[r_{1}^{o}M_{1}^{2}\left(m_{1}-1\right)\right]}_{i}^{2}+{\left[M_{2}^{2}\right]}_{i}^{2}}+r_{2}^{o}\sum \frac{2{\left[M_{2}^{2}\right]}_{i}^{2}}{{\left[r_{1}^{o}M_{1}^{2}\left(m_{1}-1\right)\right]}_{i}^{2}+{\left[M_{2}^{2}\right]}_{i}^{2}}+\sum \frac{2{\left[M_{2}^{2}\right]}_{i}{\left[M_{1}M_{2}\left(2m_{1}-1\right)\right]}_{i}}{{\left[r_{1}^{o}M_{1}^{2}\left(m_{1}-1\right)\right]}_{i}^{2}+{\left[M_{2}^{2}\right]}_{i}^{2}}=0.$$

Solving the Equations (17) and (18) can obtain the optimal values of reactivity ratios *r*_1_^0^, *r*_2_^0^.

The algorithm described above is a k nearest neighbour (k-NN) regression algorithm where k = n, where the differential copolymerization equation is used. The algorithm called k nearest neighbour [11] (k-NN) is a non-parametric regression algorithm that is permitted to obtain an optimal point based on calculation of the Euclidian distance between k points located in neighbourhood, where k is an integer chosen value between 2 and total number of points of data set.

An approach in determining the reactivity ratios is either the linearization of the Mayo-Lewis differential Equation (5) or the linearization of the integral Equation (9). The method proposed by Fineman and Ross [12] is chronologically the first method that uses the linearization of the Mayo–Lewis differential Equation (5). The mathematical equations that describe the method proposed by Fineman and Ross are:

(19) $$\frac{F}{f}\left(f-1\right)=r_{1}\frac{F^{2}}{f}-r_{2};$$

(20) $$\frac{f-1}{F}=-r_{2}\frac{f}{F^{2}}+r_{1},$$

where,

(21) $$f=\frac{m_{1}}{m_{2}}\approx \frac{dM_{1}}{dM_{2}}\;\;\;\mathrm{and}\;\;\;\;F=\frac{M_{1}}{M_{2}}.$$

Equation (19) is known as the Fineman–Ross method (FR) and Equation (20) as the reverse Fineman–Ross method (r-FR).

The disadvantage of the uneven distribution of points along the line passing between the calculated points, which is observed in the Fineman–Ross method, was removed by Kelen–Tudos [13,14] (KT) by using a correction factor α which is calculated with the relation:

(22) $$\alpha =\sqrt{F_{min}\cdot F_{max}},$$

where

(23) $$F=\frac{x^{2}}{y};$$

(24) $$x=\frac{M_{1}}{M_{2}}\;\;\;\;\;\;\mathrm{and}\;\;\;\;\;\;\;y=\frac{dM_{1}}{dM_{2}}\approx \frac{m_{1}}{m_{2}}.$$

Considering this aspect presented above, the Mayo-Lewis Equation (5) is rewritten in the form:

(25) $$\frac{G}{\alpha +F}=\left(r_{1}+\frac{r_{2}}{\alpha }\right)\frac{F}{\alpha +F}-\frac{r_{2}}{\alpha }$$

where

(26) $$G=x\frac{y-1}{y}\;.$$

In the coordinate system *G*/(*α* + *F*), *F*/(*α* + *F*) the points calculated by means of the Equation (25) have a uniform and collinear distribution.

The KT linear method has been extended to be used to determine reactivity ratios for experimental data obtained at high conversions [15] (e-KT). In this case Equation (25) is rewritten as follows:

(27) $$\frac{z\left(y-1\right)}{\alpha z^{2}+y}=\left(r_{1}+\frac{r_{2}}{\alpha }\right)\frac{y}{\alpha z^{2}+y}-\frac{r_{2}}{\alpha }\;,$$

where

(28) $$z=\frac{\log\frac{M_{1}}{M_{10}}}{\log\frac{M_{2}}{M_{20}}}=\frac{\log\left[1-\frac{y}{x_{0}}\log\left(1-P_{n}\frac{\bar{\alpha }+x_{0}}{\bar{\bar{\alpha }}+y}\right)\right]}{\log\left(1-P_{n}\frac{\bar{\alpha }+x_{0}}{\bar{\bar{\alpha }}+y}\right)}\;;$$

(29) $$\bar{\alpha }=\frac{\mu_{1}}{\mu_{2}}\;;$$

(30) $$x_{0}=\frac{M_{10}}{M_{20}}\;\mathrm{and}\;\;\;\;\;y=\frac{m_{1}}{m_{2}}\;,$$

where the 0 index refer to the initial concentration of monomer *i*, *α* has the same mathematical form as presented above, *P_n_* weight percent conversion, *μ*—molecular weight of monomers.

For all the linear methods presented above we can write a generalized equation of the following form:

(31) ζ=aη+b,

where *ζ*-dependent variable, *η*-independent variable, *a*—slope, *b*—intercept. The line parameters for the methods presented above are centralized in Table 1.

Determination of the slope (*a*) and the intercept (*b*) (31) for a line can be obtained using the ordinary least squares methods (OLS) described by following relations:

(32) $$a=\frac{n\cdot \sum \left(\eta_{i}\cdot \zeta_{i}\right)-\sum \eta_{i}\cdot \sum \zeta_{i}}{n\cdot \sum \eta_{i}^{2}-{\left(\sum \eta_{i}\right)}^{2}}\;,$$

(33) $$b=-\frac{\sum \eta_{i}^{2}\cdot \sum \zeta_{i}-\sum \eta_{i}\cdot \sum \left(\eta_{i}\cdot \zeta_{i}\right)}{n\cdot \sum \eta_{i}^{2}-{\left(\sum \eta_{i}\right)}^{2}}\;.$$

Using OLS to obtain the best slope and intercept values, the parameters *ζ* and *η* must respect the Gauss–Markov assumptions, which are:

(a) The independent variable *η* must not be correlated with the dependent variable *ζ*. This is the fundamental hypothesis of OLS. By linearization of the Mayo–Lewis Equation (5) the obtained parameters *ζ* and *η* have a degree of correlation, this fact leads to obtaining erroneous or inconsistent values for *a* and *b* parameters
(b) The non-linearity between *ζ* and *η* parameters, and if the errors are not random gives wrong estimation of *a* and *b* parameters.
(c) The estimation of the *a* and *b* parameters values is less accurate if the covariance of the errors of *η* is not constant. The covariance of errors of the parameter *η* represents a measure of the uncertainty of the model.
(d) The intercept value (*b*) is biased if the expected error in terms of the independent variable *η* is not zero
(e) All calculated values for the *η* parameter obtained by using the linear forms of the Mayo–Lewis Equation (5) must be collinear, otherwise the values of *a* and *b* parameters obtained by using the OLS method will be have big errors.

Therefore, obtaining reactivity ratios by linearizing the Mayo–Lewis Equation (5) is limiting because it is difficult to fully respect Gauss–Markov’s assumptions.

Considering the above, Tidwell and Mortimer [16] approached the solution of the Mayo–Lewis Equation (5) through a nonlinear view. Tidwell and Mortimer (TM) derived the Mayo–Lewis equation written in the form proposed by Wall [4] and Skeist [5] (6) obtaining the following relation:

(34) $$m_{2_{i}}^{j}=G_{i}^{j}+\left(r_{1}^{0}+r_{1}^{j}\right)\frac{\partial G_{i}^{j}}{\partial r_{1}}+\left(r_{2}^{0}+r_{2}^{j}\right)\frac{\partial G_{i}^{j}}{\partial r_{2}}+\varepsilon_{i}\;,$$

where:

(35) $$G^{j}=\frac{r_{2}^{j}f_{2}^{2}+f_{1}f_{2}}{r_{2}^{j}f_{2}^{2}+2f_{1}f_{2}+r_{1}^{j}f_{1}^{2}}\;,$$

*i* is the number of the experimental run, j is number of the estimation set and $r_{1}^{0}$, $r_{2}^{0}$ are the expectation values of $r_{1}^{j}$ and $r_{2}^{j}$ respectively.

By making the difference (d) between the measured value of the composition of the copolymer ($m_{2_{i}}^{j}$) and the calculated composition of the copolymer ($G^{j}$), the following equation is obtained:

(36) $$d_{i}=m_{2_{i}}^{j}-G_{i}^{j}=\beta_{1}\frac{\partial G_{i}^{j}}{\partial r_{1}}+\beta_{2}\frac{\partial G_{i}^{j}}{\partial r_{2}}+\varepsilon_{i}\;,$$

then estimates, ${\hat{\beta }}_{1}$,$\;{\hat{\beta }}_{2}$ of the smallest squares of $\beta_{1}$ and $\beta_{2}$ provide the necessary corrections so that the new values of $r_{1}^{j}$ and $r_{2}^{j}$ given by:

(37) $$r_{1}^{j+1}=r_{1}^{j}+\beta_{1}\;,$$

(38) $$r_{2}^{j+1}=r_{2}^{j}+\beta_{2}\;.$$

The method proposed by Tidwell and Mortimer uses the Gauss–Newton optimization algorithm by minimizing $\sum {\left(d_{i}\right)}^{2}$ for the search for the best pair of reactivity ratios.

It is well known that any experimental measurement contains errors, and for this reason a number of authors [17,18,19,20,21,22,23,24,25,26] have used the principle of minimizing these errors to obtain the true value of composition of the feed and the copolymer, and finally to obtain the best values of reactivity ratios.

This concept, called error in variable method (EVM), was originally developed by German [17] considering the error in only one variable. Later van der Meer et al. [18] extended the concept to analysis the errors in both variables, after which various approaches appeared in the calculation methodology [19,20,21,22,23,24,25,26]. For the comparative analysis of the methods for calculating the reactivity ratios, the EVM variant proposed by Chee and Ng [26] was chosen, because it uses the integral equation proposed by Mayo and Lewis (12) and does not require to know the experimental error.

The variant of EVM proposed by Chee and Ng (EVM-CN) minimizes the objective function given by the relationship:

(39) $$S=\sum W{\left(r_{2}-r_{2}^{e}\right)}^{2}$$

where

(40) $$W=\frac{1}{Var\left(r_{2}-r_{2}^{pe}\right)}=\frac{1}{Var\left(f\right)}$$

(41) $$Var\left(f\right)={\left(\frac{\partial f}{\partial x}\right)}^{2}Var\left(x\right)+{\left(\frac{\partial f}{\partial y}\right)}^{2}Var\left(y\right)+{\left(\frac{\partial f}{\partial P_{n}}\right)}^{2}Var\left(P_{n}\right)+2\left(\frac{\partial f}{\partial x}\right)\left(\frac{\partial f}{\partial y}\right)Cov\left(x,y\right)+2\left(\frac{\partial f}{\partial y}\right)\left(\frac{\partial f}{\partial P_{n}}\right)Cov\left(y,P_{n}\right)+2\left(\frac{\partial f}{\partial x}\right)\left(\frac{\partial f}{\partial P_{n}}\right)Cov\left(x,P_{n}\right)$$

(42) $$x=\frac{M_{10}}{1-M_{10}}\;\;\;\;\;\;\;\;\;\;\;\;\;\;y=\frac{m_{1}}{1-m_{1}}$$

(43) $$Var\left(x\right)={\left(1+x\right)}^{4}\sigma_{M}^{2}$$

(44) $$Var\left(y\right)={\left(1+y\right)}^{4}\sigma_{M}^{2}\;$$

(45) $$Var\left(P_{n}\right)=P_{n}\left\{{\left(\frac{\sigma_{P}}{P_{w}}\right)}^{2}+{\left(1-\bar{\alpha }\right)}^{2}\left[{\left(\frac{x}{1+\bar{\alpha }x}\right)}^{2}{\left(\frac{\sigma_{M}}{M_{10}}\right)}^{2}+{\left(\frac{y}{1+\bar{\alpha }y}\right)}^{2}{\left(\frac{\sigma_{m}}{M_{1}}\right)}^{2}\right]\right\}$$

(46) Cov(x,y)=0

(47) $$Cov\left(y,P_{n}\right)=\left(\frac{\partial P_{n}}{\partial y}\right)Var\left(y\right)$$

(48) $$Cov\left(x,P_{n}\right)=\left(\frac{\partial P_{n}}{\partial x}\right)Var\left(x\right)$$

$r_{2}^{e}$—the value of r_2_ estimated with Equation (12), *P_n_*—weight percent conversion, *σ*—standard deviation of *M*_10_, *m*_1_—molar fraction of monomer 1 in copolymer.

Although the methods for calculating reactivity ratios using the EVM technique are integral methods, they do not include conversion measurement errors in their analysis.

The non-parametric regression algorithm k-NN is widely used in medicine and pharmaceutics [27,28,29,30,31], machine learning [32,33,34,35], the facial recognition algorithm programs [36], traffic flow prediction [37] and many other fields.

The new integral method proposed below is an adaptation of the non-parametric k-NN regression algorithm to the calculation of reactivity ratios from terminal model of binary copolymerization.

## 2. Materials and Methods

In the work of Mayo and Lewis [3] the following expression draws attention, “The experimental error, measured by the size of the area bounded by the three lines, is halved by a change of only 0.10% in the carbon analysis (0.5% in the styrene content) of the copolymer”.

In the coordination system *r*_1_, *r*_2_ through the intersection of three lines results a triangle whose vertices are described by the coordinates of the points *P^i^* (*r*_1_, *r_2_*), *P^j^* (*r*_1_, *r*_2_) and *P^q^* (*r_1_*, *r*_2_). The determination of the values of the coordinates of the points *P^i^* (*r*_1_, *r*_2_), *P^j^* (*r*_1_, *r*_2_) and *P^q^* (*r*_1_, *r*_2_) is undertaken by solving the following system of equations:

(49) $$\{\begin{array}{c} r_{2}^{i}=a_{i}r_{1}^{i}+b_{i}\\ r_{2}^{j}=a_{j}r_{1}^{j}+b_{j}\\ r_{2}^{q}=a_{q}r_{1}^{q}+b_{q} \end{array}\;,$$

where:

(50) $$a_{\left(i,i,q\right)}={\left[\frac{f_{1}^{\left(i,j,q\right)}}{f_{2}^{\left(i,j,q\right)}}\right]}^{2}\cdot \frac{m_{2}}{m_{1}}\;\;\;\;\;\;\;and\;\;\;\;\;\;\;\;\;b_{\left(i,j,q\right)}=\frac{f_{1}^{\left(i,j,q\right)}}{f_{2}^{\left(i,j,q\right)}}\cdot \left(\frac{m_{2}}{m_{1}}-1\right)\;,$$

*i*, *j*, *q*—indices referring to the number of the experimental point from data set.

By solving the system of Equation (49) for “n” experimental points a number of “m” of triangles can be generated, according with the relation (51):

(51) $$m=C_{n}^{3}=\frac{n\cdot \left(n-1\right)\cdot \left(n-2\right)}{6}$$

The calculation of the experimental errors starting from the statement of Mayo and Lewis [3] is undertaken by solving the following system of Equation (52):

(52) $$\{\begin{array}{c} S_{1}=\varepsilon_{1}^{1}+\varepsilon_{2}^{1}+\varepsilon_{3}^{1}+\varepsilon_{4}^{1}+\cdots +\varepsilon_{n}^{1}\\ \begin{array}{c} S_{2}=\varepsilon_{1}^{2}+\varepsilon_{2}^{2}+\varepsilon_{3}^{2}+\varepsilon_{4}^{2}+\cdots +\varepsilon_{n}^{2}\\ \begin{array}{c} \begin{array}{c} \vdots \\ S_{i}=\varepsilon_{1}^{i}+\varepsilon_{2}^{i}+\varepsilon_{3}^{i}+\varepsilon_{4}^{i}+\cdots +\varepsilon_{n}^{i} \end{array}\\ \begin{array}{c} \vdots \\ S_{m}=\varepsilon_{1}^{m}+\varepsilon_{2}^{m}+\varepsilon_{3}^{m}+\varepsilon_{4}^{m}+\cdots +\varepsilon_{n}^{m} \end{array} \end{array} \end{array} \end{array}$$

where *S_i_*—the size of area of the triangle, *i* = 1… m; $\varepsilon_{j}^{i}$, $\varepsilon_{q}^{i}$, $\varepsilon_{s}^{i}$—the errors of the experiments that leads to the formation of the triangle *i*.

The surface of the formed triangle, where are knowing the values of its peaks *P^i^* (*r*_1_, *r*_2_), *P^j^* (*r*_1_, *r*_2_) and *P^q^* (*r*_1_, *r*_2_) is calculated with the following relation (53):

(53) $$S_{i}=r_{1}^{i}r_{2}^{j}+r_{1}^{q}r_{2}^{i}+r_{1}^{j}r_{2}^{q}-r_{1}^{q}r_{2}^{j}-r_{1}^{i}r_{2}^{q}-r_{1}^{j}r_{2}^{i}$$

The solutions of the system of Equations (52) are obtained by solving the matrix Equation (54):

(54) $$\left(\begin{array}{ccccccccc} 1 & 1 & 1 & 0 & 0 & \cdots & 0 & 0 & 0\\ 1 & 1 & 0 & 1 & 0 & \cdots & 0 & 0 & 0\\ \vdots & & & & & & & & \\ 1 & 1 & 0 & 0 & 0 & \cdots & 0 & 0 & 1\\ \vdots & & & & & & & & \\ 0 & 1 & 1 & 1 & 0 & \cdots & 0 & 0 & 0\\ 0 & 1 & 1 & 0 & 1 & \cdots & 0 & 0 & 0\\ 0 & 1 & 1 & 0 & 0 & & 0 & 0 & 1\\ \vdots & & & & & & & & \\ 0 & 0 & 0 & 0 & 0 & \cdots & 1 & 1 & 1 \end{array}\right)\cdot \left(\begin{array}{c} \varepsilon_{1}\\ \varepsilon_{2}\\ \vdots \\ \varepsilon_{i}\\ \vdots \\ \varepsilon_{j}\\ \varepsilon_{j+1}\\ \varepsilon_{j+2}\\ \vdots \\ \varepsilon_{m} \end{array}\right)=\left(\begin{array}{c} S_{1}\\ S_{2}\\ \vdots \\ S_{i}\\ \vdots \\ S_{j}\\ S_{j+1}\\ S_{j+2}\\ \vdots \\ S_{m} \end{array}\right)$$

Based on these observations presented above, it was considered that the determination of reactivity ratios could be achieved by an error regression analysis using the k-NN algorithm where k = 3. The method of calculating the reactivity ratios using the k-NN regression algorithm has the following steps:

1. Calculate all possible sets of *P*_3_*^t^* (*r*_1_, *r*_2_) points that can be generated from the experimental data set.
2. For each set of points, *P*_3_*^t^* (*r*_1_, *r*_2_) will calculate the weight center, *P^cen^* (${\hat{r}}_{1}^{j}$, ${\hat{r}}_{2}^{j}$), using the relations:
  (55) $${\hat{r}}_{1}^{j}=\frac{1}{3}\cdot \overset{3}{\underset{i=1}{\sum }}r_{1}^{i,j}$$
  (56) $${\hat{r}}_{2}^{j}=\frac{1}{3}\cdot \overset{3}{\underset{i=1}{\sum }}r_{2}^{i,j}$$
  where ${\hat{r}}_{1}^{j}$, ${\hat{r}}_{2}^{j}$—the coordinates of the weight center of a set of points *P*_3_*^t^* (*r*_1_, *r*_2_), *r*_1_*^i,j^*, *r*_2_*^i,j^*—the coordinates of the vertices of the triangle in the data set *P*_3_*^t^*(*r*_1_, *r*_2_), i—index of the vertices point *i* = 1,2,3 and *j*—number of set of points *P*_3_*^t^*(*r*_1_, *r*_2_), $j_{max}=\frac{\left(n-1\right)\left(n-2\right)}{2}$ where n—number of experimental data sets.
3. For each *P^cen^*(${\hat{r}}_{1}^{j}$, ${\hat{r}}_{2}^{j}$) point, calculate the composition of the substrate using the integration method [24,25] until the experimental conversion of each point from the experimental data set is touched.
4. Using the experimental data of copolymer composition and calculated copolymer composition with ${\hat{r}}_{1}^{j}$, ${\hat{r}}_{2}^{j}$, calculate the value of the objective function, the Fischer criterion (*F^c^*) [38], using the relation (57)
  (57) $$F_{j}^{c_{cen}}=\sqrt{\frac{\sum_{j=1}\sum_{i=1,2}{\left(m_{i}^{j\left(e\right)}-m_{i}^{j\left(c\right)}\right)}^{2}}{n\left(p-n+1\right)}}$$
  where $F_{j}^{c_{cen}}$ is the value of the Fisher criterion for the reactivity ratios from the center of each triangle, *n* is the number of monomers used in copolymerization and *p* is the number of the experimental data set. Thus, *m_i_^j(e)^* is the molar fraction of monomer “*i*” from copolymer for “*j*” experimental data set, *m_i_^j(c^*^)^ is the molar fraction of monomer “*i*” calculated based on a mathematical model for the experiment “*j*”.
5. The *P^cen^(*${\hat{r}}_{1}^{j}$, ${\hat{r}}_{2}^{j}$) points are ordered in ascending order according to the value of $F_{j}^{c_{cen}}$ at which point is selected the first n points *P^cen^(*${\hat{r}}_{1}^{j}$, ${\hat{r}}_{2}^{j}$*)* which have the lowest $F_{j}^{c_{cen}}$ values. These selected points will generate a new set of points *P*_3_*^t^* (*r*_1_, *r*_2_). This step is intended to eliminate the reactivity ratios which have great errors and to reduce computation time.
6. The error of the optimization process is evaluated with the following relation:
  (58) $$err=\left|1-\frac{F_{1}^{c_{s}}}{F_{1}^{c_{s-1}}}\right|$$
  where $F_{1}^{c_{s}}$—the best value of Fischer criterion at step *s* of the optimization process, $F_{1}^{c_{s-1}}$—the best value of Fischer criterion at step *s* − 1 of the optimization process. If the error (err) is not less than 1 × 10^−4^, then with the last generated set of points *P*_3_*^t^* (*r*_1_, *r*_2_) return to step 2, else the search process will be stopped.

The reactivity ratios which have the lowest value of the Fischer criterion from the last search step will become the final solution of the optimization process.

In order to verify the quality of the new method compared to the methods presented above, an analysis plan was drawn up on simulated data in which the chosen reactivity ratios must meet the conditions: *r*_1_ × *r*_2_ ≈ 0, *r*_1_ × *r*_2_ ∊ [0.5,1], *r*_1_ × *r*_2_ > 1. Table 2 shows the data of a comparison of the quality analysis plan for a new method with the most used methods in reactivity ratios determination, presented above.

The reactivity ratios were chosen randomly in such a way as to meet the conditions imposed above. The feed composition and the conversions were obtained by a normalized randomly software. The copolymer composition was obtained by numerical integration until the specific conversion of each point was reached. Moreover, the methods presented above were also verified on real experimental data for copolymerization of:

a. 2-(*N*-phthalimido) ethyl acrylate (NPEA) with 1-vinyl-2-pyrolidone (NVP), initiated by AIBN in DMF at 70 °C [39];
b. Isoprene (Is) with glycidyl methacrylate (GMA), initiated by AIBN in bulk at 70 °C [40];
c. *N*-isopropylacrylamide (NIPAM) with *N*,*N*-dimethylacrylamide (DMA), initiated by AIBN in DMF at 70 °C [41].

The simulated input data, which were used in the comparative qualitative analysis of the methods for calculating the reactivity ratios presented above are shown in Table 3, Table 4, Table 5, Table 6, Table 7, Table 8, Table 9, Table 10 and Table 11. The estimated errors shown in the tables below are obtained by solving Equation (39) for given data.

The software used to determine the reactivity ratios with the methods described above was coded in Python 3.

## 3. Results

The reactivity ratios obtained in this analysis, as well as the Fisher criterion values, using the input from Table 3, Table 4, Table 5, Table 6, Table 7, Table 8, Table 9, Table 10 and Table 11, are presented in Table 12, Table 13, Table 14, Table 15, Table 16, Table 17, Table 18, Table 19 and Table 20. In these tables, the reactivity ratios obtained by the methods used in this analysis are ascending, ordered according to the value of the Fisher criterion (*F^c^*), and the bias represents the value of the difference from the calculated value of the reactivity ratios and the imposed target value.

To highlight the way in which the integral method k-NN looks for the best point, Figure 1, Figure 2, Figure 3, Figure 4, Figure 5, Figure 6, Figure 7, Figure 8 and Figure 9 present the points *P^cen^* (*r*_1_, *r*_2_) obtained for each search step, where the best point represent the final solution of k-NN method.

For a complete analysis of the quality of the k-NN method and the other methods used in this comparative analysis, the 95% confidence domains (JCR) were plotted for all nine imposed conditions. Relation (59) was used to trace these JCRs:

(59) $$S\left(\theta \right)-S\left(\hat{\theta }\right)\leq ps^{2}F\left(p,n-p,\alpha \right)$$

where,

(60) $$S\left(\hat{\theta }\right)={\left[y_{i}-f({\underline{x}}_{i},\underline{\hat{\theta }}\right]}^{T}\left[y_{i}-f({\underline{x}}_{i},\underline{\hat{\theta }}\right]$$

**Figure 10:**
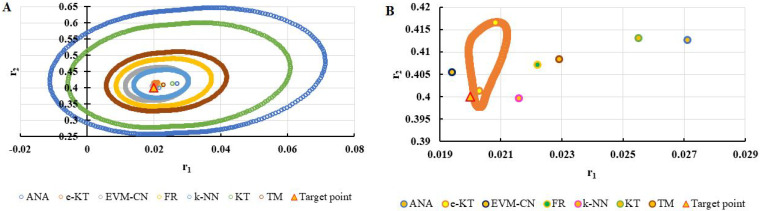
The JCR of analyzed methods for reactivity ratios calculation for LC1 imposed condition (**A**) and detail of smallest JCR with distribution of reactivity ratios (**B**).

**Figure 11:**
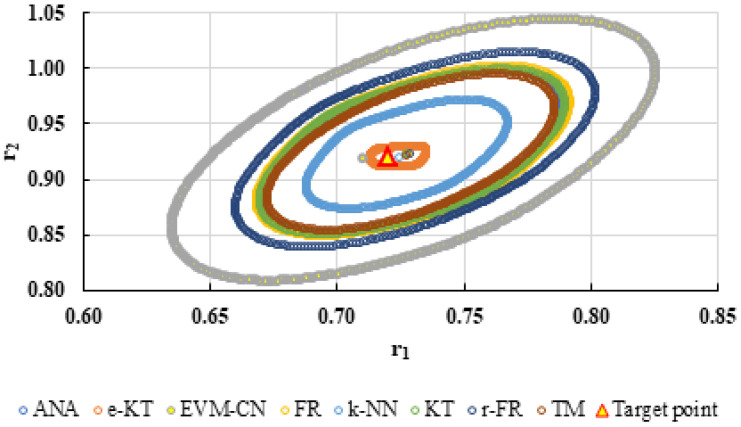
The JCR of analyzed methods for reactivity ratios calculation for LC2 imposed condition.

**Figure 12:**
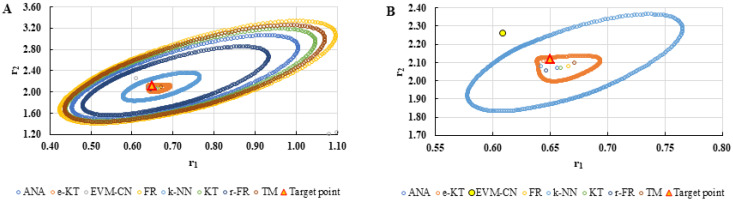
The JCR of analyzed methods for reactivity ratios calculation for LC3 imposed condition (**A**) and detail of smallest JCR with distribution of reactivity ratios (**B**).

**Figure 13:**
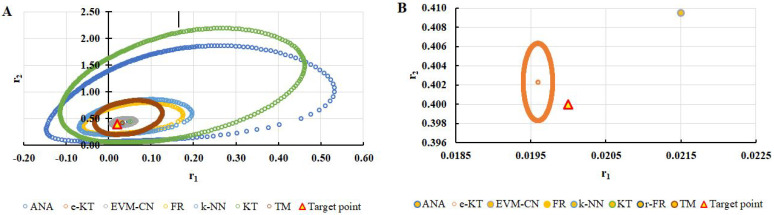
The JCR of analyzed methods for reactivity ratios calculation for MC1 imposed condition (**A**) and detail of smallest JCR with distribution of reactivity ratios (**B**).

**Figure 14:**
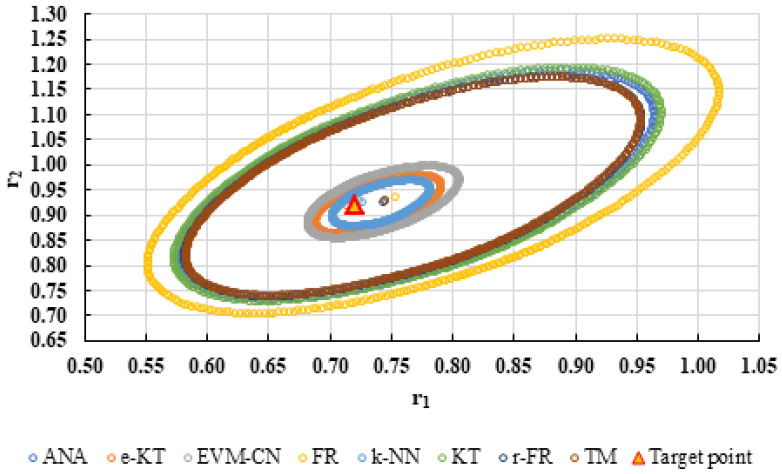
The JCR of analyzed methods for reactivity ratios calculation for MC2 imposed condition.

**Figure 15:**
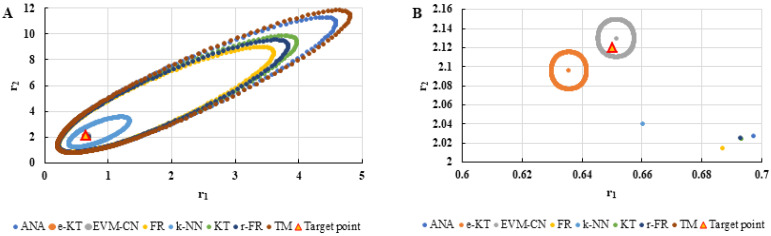
The JCR of analyzed methods for reactivity ratios calculation for MC3 imposed condition (**A**) and detail of smallest JCR with distribution of reactivity ratios (**B**).

**Figure 16:**
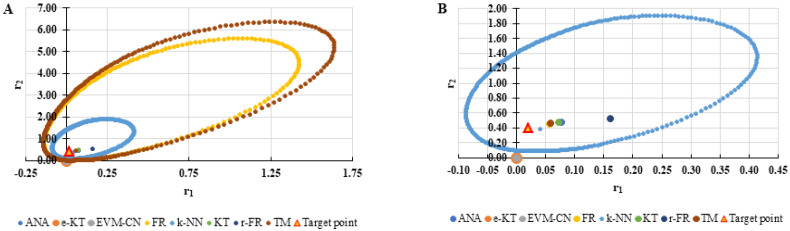
The JCR of analyzed methods for reactivity ratios calculation for HC1 imposed condition (**A**) and detail of smallest JCR with distribution of reactivity ratios (**B**).

**Figure 17:**
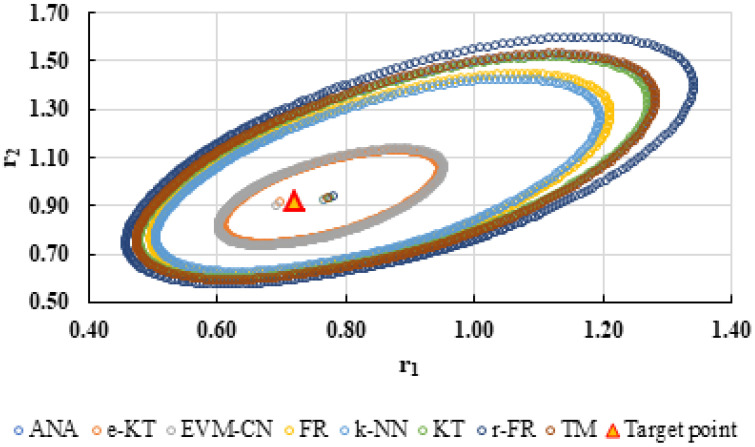
The JCR of analyzed methods for reactivity ratios calculation for HC2 imposed condition.

**Figure 18:**
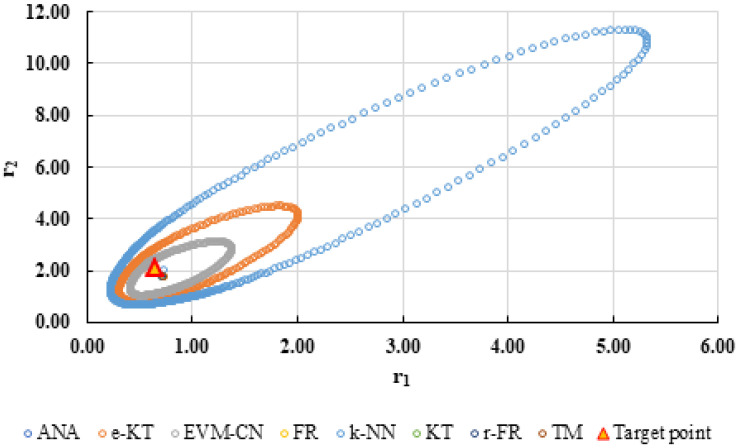
The JCR of analyzed methods for reactivity ratios calculation for HC3 imposed condition.

Equation (59) was defined by Mathew and Duever as the “exact shape” of JCR [42]. In Figure 10, Figure 11, Figure 12, Figure 13, Figure 14, Figure 15, Figure 16, Figure 17 and Figure 18, the JCRs that do not appear in the graph are so large that they would make the small ones no longer visible. In the following figures, the target value represent the chosen reactivity ratios for each simulated experiment.

**Figure 19:**
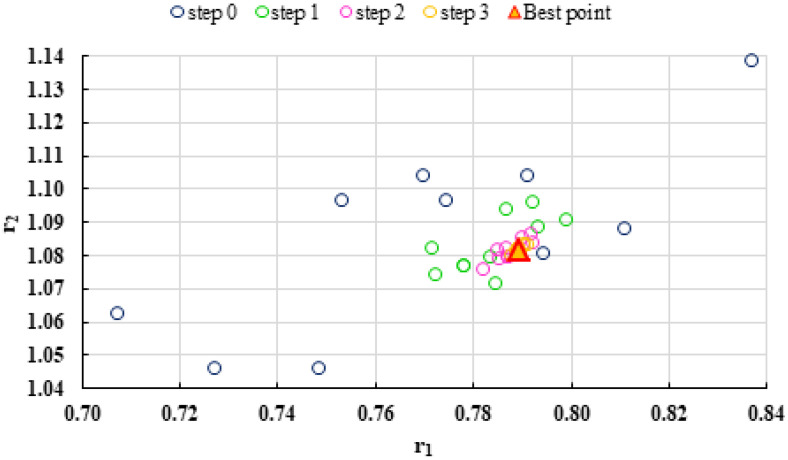
Distribution of *P^cen^(r_1_, r_2_)* points for each step of searching for the k-NN method for copolymerization of 2-(*N*-phthalimido) ethyl acrylate with 1-vinyl-2-pyrolidone (NPEA-NVP) [39].

**Figure 20:**
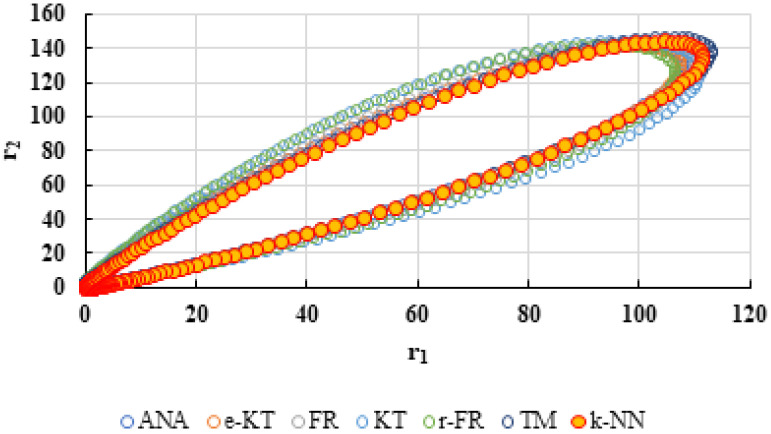
The JCR of analyzed methods for reactivity ratios calculation for copolymerization of NPEA-NVP [39].

**Figure 21:**
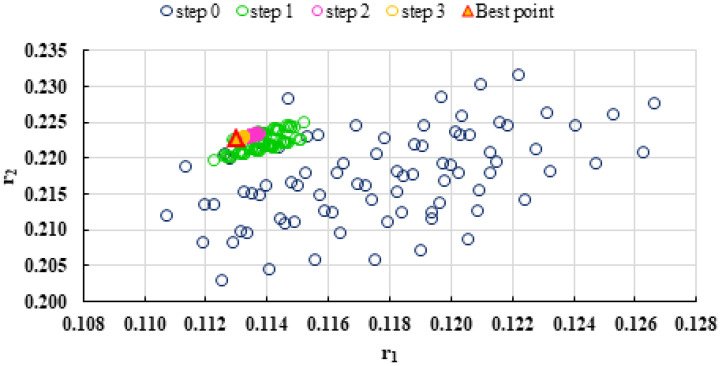
Distribution of *P^cen^*(*r*_1_, *r*_2_) points for each step of searching for the k-NN method for copolymerization of Is-GMA [40].

**Figure 22:**
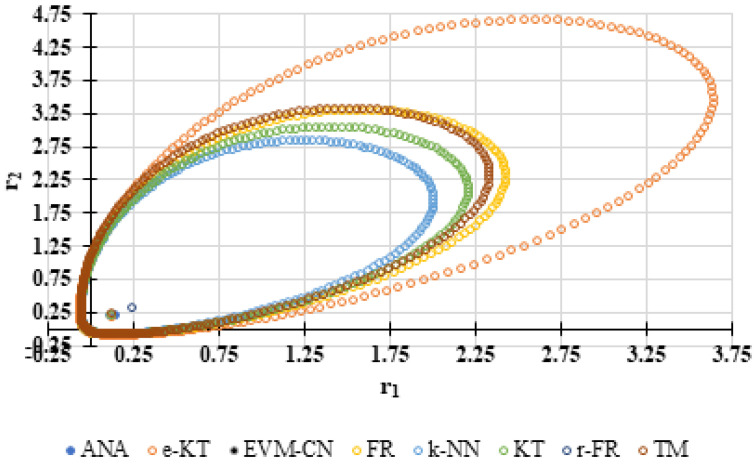
The JCR of analyzed methods for reactivity ratios calculation for copolymerization of Is-GMA [40].

**Figure 23:**
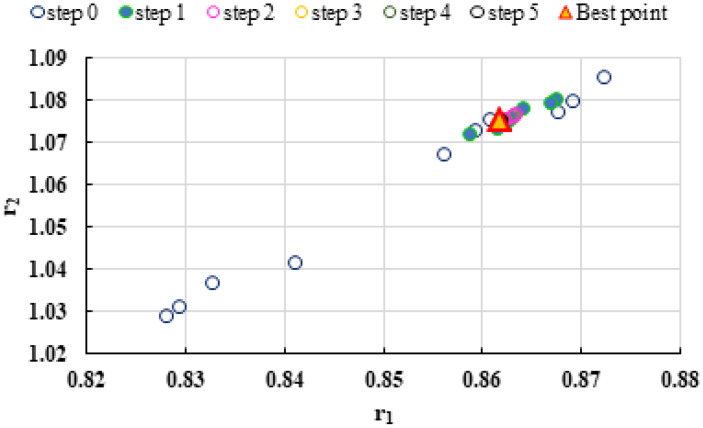
Distribution of *P^cen^(r_1_, r_2_)* points for each step of searching for the k-NN method for copolymerization of NIPAM-NVP [41].

**Figure 24:**
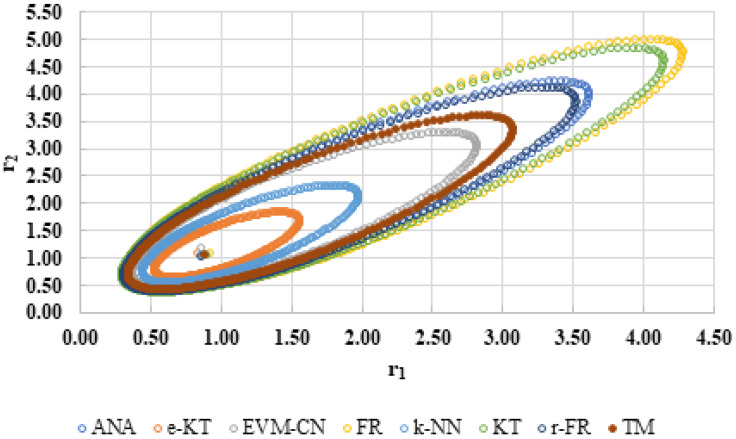
The JCR of analyzed methods for reactivity ratios calculation for copolymerization of NIPAM-NVP [41].

The k-NN method for determining the reactivity ratios proposed in this paper as well as the other methods used in this comparative analysis were also tested on real experimental data. The results obtained are presented in Table 21, Table 22 and Table 23 and Figure 19, Figure 20, Figure 21, Figure 22, Figure 23 and Figure 24.

## 4. Discussion

The visualization of the search steps of the k-NN method shows us that the elimination of the pairs of irrelevant reactivity ratios using as criterion of elimination the value of *F^c^* not only increases the calculation speed but also improves the quality of the result. The improvement in the quality of the result is given by the fact that the numerator of the function *F^c^* is in fact a residual variation due to errors (61).

(61) $$\sum {\left(m_{1}^{j\left(e\right)}-m_{1}^{j\left(c\right)}\right)}^{2}=\sum \varepsilon_{j}^{2}$$

The results from Table 13, Table 14, Table 15, Table 16, Table 17, Table 18, Table 19, Table 20 and Table 21 show that the integral k-NN method is in good agreement with the other integral methods for determining reactivity ratios and obviously better than the differential methods used in this comparative analysis.

On the other hand, if we corroborate the data from Table 13, Table 14, Table 15, Table 16, Table 17, Table 18, Table 19, Table 20 and Table 21 with the JCRs presented in Figure 10, Figure 11, Figure 12, Figure 13, Figure 14, Figure 15, Figure 16, Figure 17 and Figure 18, it is observed that:

- The e-KT method has the lowest values of *F^c^* in the case of the conditions imposed by LC1, LC2, LC3, MC1 and HC2 but at the same time the target value imposed for LC1, MC1 and MC3 is outside the JCR determined for this method. Taking into account that JCR represents the set of reactivity ratios that are solutions of the method with 95% confidence and the target value is not part of these solutions, the e-KT method cannot be considered the best method in the situations presented above.
- The EVM-CN method is the best method for the MC3 and HC3 conditions.
- The reactivity ratios obtained by the EVM-CN method for the imposed conditions LC2, and LC3 are outside the JCR of the best method for these cases.
- Under the conditions imposed by HC1, the e-KT and EVM-CN methods did not give good results because the calculation method uses logarithms whose argument takes negative values for large conversions and appropriate reactivity ratios of 0.

The true value of the k-NN method is demonstrated by the results obtained on real experimental data which proves that it is a solid method and can be used successfully at any conversion of less than 55%.

## 5. Conclusions

The integral method for determining the reactivity ratios based on the k-NN regression algorithm proposed in this paper is a simple method based on the intersection method. The k-NN method provides results comparable to any other integral method. The k-NN method is stable for any combination of reactivity ratios and can be used successfully up to 55% conversions. The notable disadvantage of this method is that it requires a minimum of six experimental points to be effective. Also, in the search process, a way to estimate the experimental errors using a single data set was determined. We believe that future works could establish models with the three conversion parts.

## Figures and Tables

**Figure 1:**
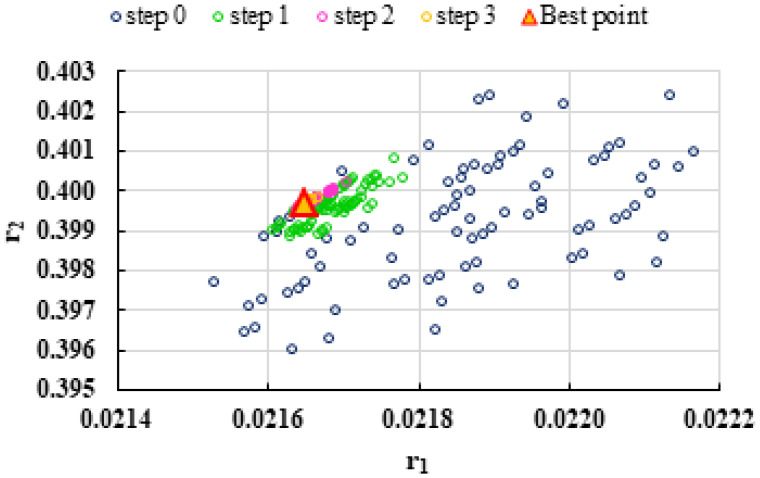
Distribution of *P^cen^*(*r*_1_, *r*_2_) points for each step of searching for the k-NN method for the conditions imposed by LC1.

**Figure 2:**
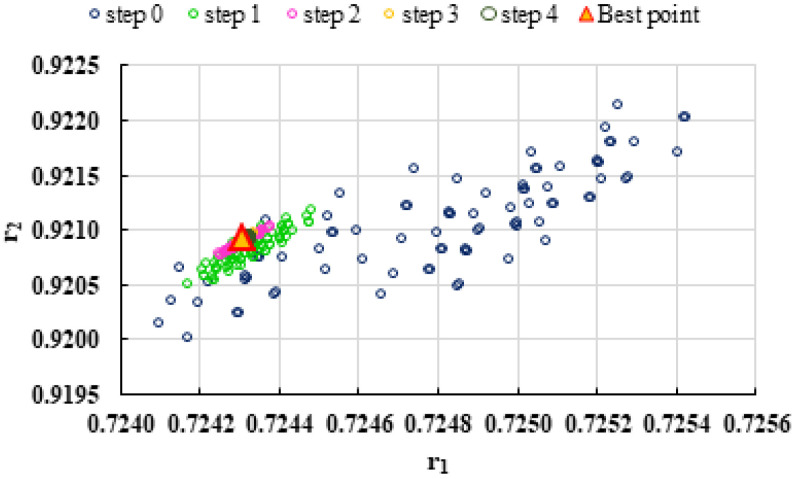
Distribution of *P^cen^*(*r*_1_, *r*_2_) points for each step of searching for the k-NN method for the conditions imposed by LC2.

**Figure 3:**
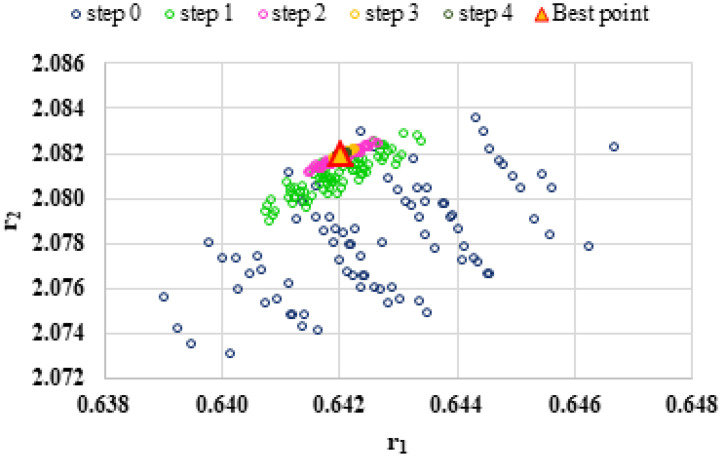
Distribution of *P^cen^*(*r*_1_, *r*_2_) points for each step of searching for the k-NN method for the conditions imposed by LC3.

**Figure 4:**
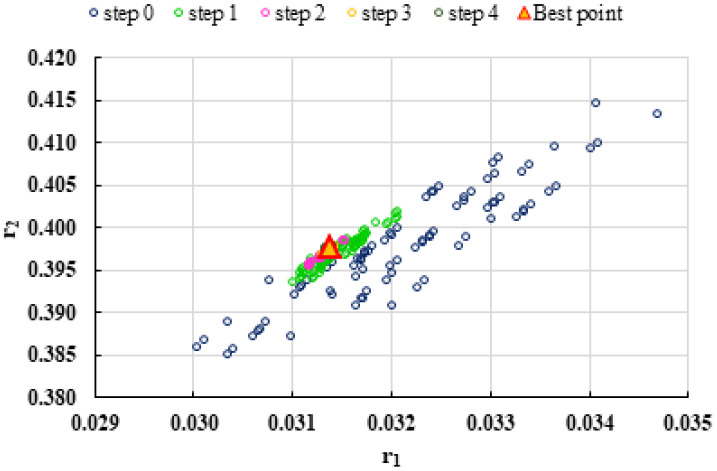
Distribution of *P^cen^*(*r*_1_, *r*_2_) points for each step of searching for the k-NN method for the conditions imposed by MC1.

**Figure 5:**
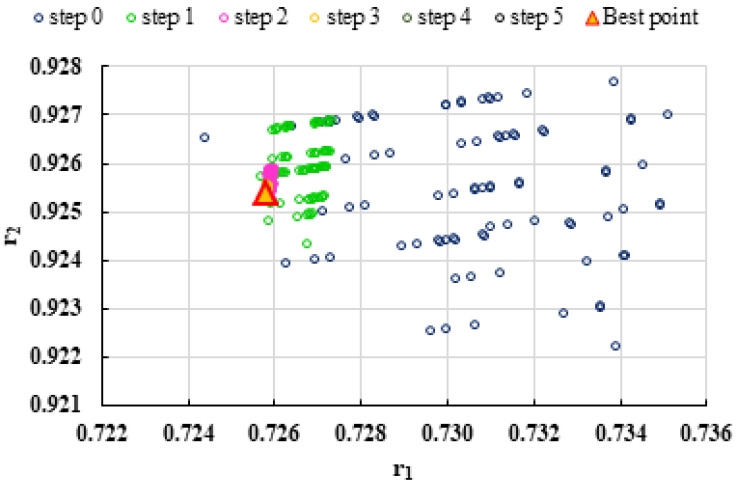
Distribution of *P^cen^*(*r*_1_, *r*_2_) points for each step of searching for the k-NN method for the conditions imposed by MC2.

**Figure 6:**
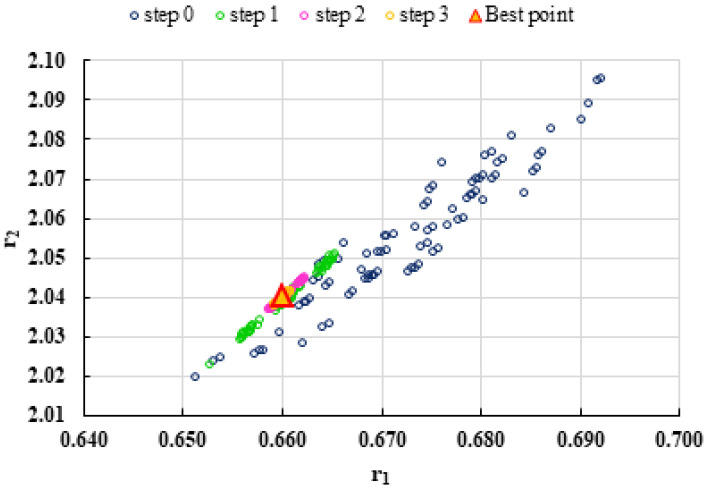
Distribution of *P^cen^*(*r*_1_, *r*_2_) points for each step of searching for the k-NN method for the conditions imposed by MC3.

**Figure 7:**
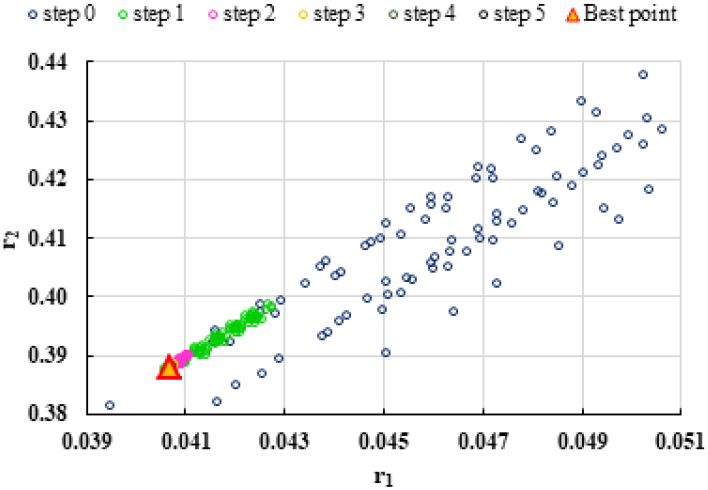
Distribution of *P^cen^*(*r*_1_, *r*_2_) points for each step of searching for the k-NN method for the conditions imposed by HC1.

**Figure 8:**
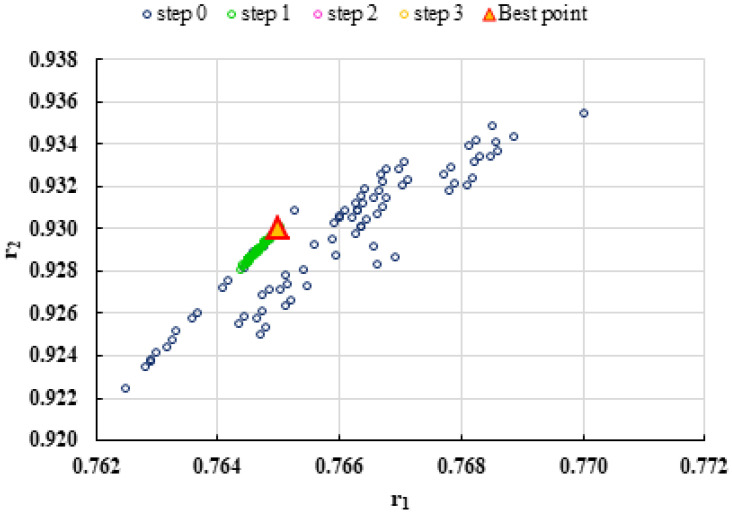
Distribution of *P^cen^*(*r*_1_, *r*_2_) points for each step of searching for the k-NN method for the conditions imposed by HC2.

**Figure 9:**
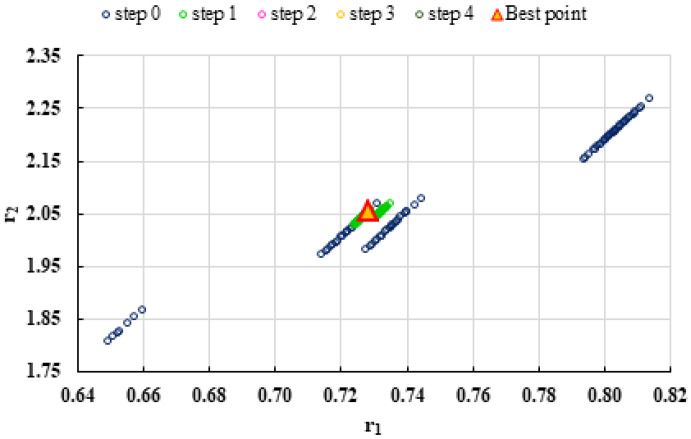
Distribution of *P^cen^*(*r*_1_, *r*_2_) points for each step of searching for the k-NN method for the conditions imposed by HC3.

**Table 1:** The line parameters for the linear methods presented above.

Method	ζ	η	a	b
FR	$\frac{F}{f}\left(f-1\right)$	$\frac{F^{2}}{f}$	*r*_1_	−*r*_2_
r-FR	$\frac{f-1}{F}$	$\frac{f}{F^{2}}$	−*r*_2_	*r*_1_
KT	$\frac{G}{\alpha +F}$	$\frac{F}{\alpha +F}$	$\left(r_{1}+\frac{r_{2}}{\alpha }\right)$	$-\frac{r_{2}}{\alpha }$
e-KT	$\frac{z\left(y-1\right)}{\alpha z^{2}+y}$	$\frac{y}{\alpha z^{2}+y}$	$\left(r_{1}+\frac{r_{2}}{\alpha }\right)$	$-\frac{r_{2}}{\alpha }$

**Table 2:** The conditions of the analysis plan for methods quality.

Nr. Crt	*r*_1_	*r*_2_	*r*_1_ × *r*_2_	Conversion (Pn) wt. %		
				LC	MC	HC
1	0.02	0.40	0.008	1–10	10–35	40–65
2	0.72	0.92	0.662			
3	0.65	2.12	1.378			

**Table 3:** The input data for low conversion and *r*_1_ = 0.02 and *r*_2_ = 0.40 (LC1).

*M*_1_	*m*_1_	*P_n_*	Estimated Error × 10^−5^
0.074	0.138	9.13	21.50
	0.177	9.10	14.30
0.207	0.282	7.54	12.90
0.305	0.345	2.91	8.14
0.401	0.388	5.80	0.74
0.530	0.431	8.68	1.65
0.638	0.459	6.22	1.21
0.770	0.489	4.04	1.08
0.878	0.523	5.09	1.00

**Table 4:** The input data for low conversion and *r*_1_ = 0.72 and *r*_2_ = 0.92 (LC2).

*M*_1_	*m*_1_	*P_n_*	Estimated Error’ × 10^−7^
0.043	0.045	7.46	7.074
0.133	0.136	6.49	−2.747
0.256	0.253	4.75	−0.971
0.329	0.320	7.52	36.576
0.451	0.429	3.86	8.855
0.561	0.529	5.85	0.536
0.654	0.614	4.39	61.212
0.757	0.716	6.71	12.783
0.833	0.797	7.40	14.802

**Table 5:** The input data for low conversion and *r*_1_ = 0.65 and *r*_2_ = 2.12 (LC3).

*M*_1_	*m*_1_	*P_n_*	Estimated Error’ × 10^−5^
0.1043	0.0546	8.94	68.701
0.1094	0.0573	7.99	50.012
0.2766	0.1631	6.15	27.355
0.3423	0.2099	2.02	111.033
0.4104	0.2668	4.02	3.127
0.5816	0.4341	5.46	−2.283
0.6652	0.5329	9.19	22.137
0.7586	0.6513	9.70	2.920
0.8059	0.7144	7.87	−3.359

**Table 6:** The input data for medium conversion and *r*_1_ = 0.02 and *r*_2_ = 0.40 (MC1).

*M*_1_	*m*_1_	*P_n_*	Estimated Error’ × 10^−4^
0.035	0.067	18.87	6.579
0.122	0.190	27.46	7.081
0.286	0.331	24.62	4.696
0.360	0.371	21.56	2.030
0.453	0.408	17.35	−1.037
0.521	0.432	30.38	−0.702
0.684	0.477	33.80	0.195
0.771	0.499	30.14	1.637
0.815	0.507	19.53	4.194

**Table 7:** The input data for medium conversion and *r*_1_ = 0.72 and *r*_2_ = 0.92 (MC2).

*M*_1_	*m*_1_	*P_n_*	Estimated Error’ × 10^−5^
0.083	0.087	18.56	−2.411
0.138	0.141	31.79	−0.757
0.219	0.219	14.25	−3.582
0.319	0.311	22.78	−3.185
0.486	0.462	15.82	1.923
0.527	0.500	25.20	11.072
0.602	0.568	12.79	9.490
0.707	0.668	16.15	12.025
0.835	0.803	32.65	20.686

**Table 8:** The input data for medium conversion and *r*_1_ = 0.65 and *r*_2_ = 2.12 (MC3).

*M*_1_	*m*_1_	*P_n_*	Estimated Error’ × 10^−4^
0.100	0.053	15.27	13.412
0.116	0.062	12.08	10.647
0.244	0.142	10.60	9.301
0.368	0.239	16.20	4.131
0.452	0.320	25.94	74.227
0.514	0.371	15.99	20.254
0.605	0.473	22.51	1.672
0.744	0.635	14.09	7.726
0.856	0.792	23.80	−6.405

**Table 9:** The input data for high conversion and *r*_1_ = 0.02 and *r*_2_ = 0.40 (HC1).

*M*_1_	*m*_1_	*P_n_*	Estimated Error’ × 10^−4^
0.083	0.127	49.22	84.801
0.101	0.153	45.44	51.577
0.224	0.283	42.8	27.435
0.339	0.359	49.21	4.095
0.434	0.404	45.33	−4.905
0.537	0.441	53.71	−6.280
0.665	0.476	46.72	−4.394
0.714	0.487	41.68	0.849
0.847	0.548	51.18	−3.644

**Table 10:** The input data for high conversion and *r*_1_ = 0.72 and *r*_2_ = 0.92 (HC2).

*M*_1_	*m*_1_	*P_n_*	Estimated Error’ × 10^−5^
0.101	0.104	49.83	−0.17
0.119	0.122	46.46	−1.41
0.220	0.220	45.61	−2.71
0.320	0.314	40.55	−1.87
0.427	0.413	56.19	1.72
0.569	0.544	59.21	20.37
0.642	0.610	44.32	11.99
0.713	0.680	50.56	5.91
0.824	0.793	40.98	15.25

**Table 11:** The input data for high conversion and *r*_1_ = 0.65 and *r*_2_ = 2.12 (HC3).

*M*_1_	*m*_1_	*P_n_*	Estimated Error’ × 10^−3^
0.071	0.042	50.59	−5.713
0.118	0.072	52.09	−5.619
0.293	0.201	54.60	−1.700
0.380	0.267	43.39	−1.761
0.472	0.349	40.31	32.770
0.502	0.392	57.10	38.335
0.612	0.495	40.62	−1.865
0.785	0.708	48.32	−1.852
0.808	0.744	58.73	−0.612

**Table 12:** Reactivity ratios obtained in the imposed conditions of LC1.

Method	*r*_1_	*r*_2_	*F^c^* × 1000	Bias	
				*r*_1_	*r*_2_
e-KT	0.0203	0.4014	0.2204	−0.0003	−0.0014
k-NN	0.0217	0.3997	0.8038	−0.0017	0.0003
EVM-CN	0.0194	0.4055	0.9495	0.0007	−0.0055
FR	0.0222	0.4072	1.3358	−0.0022	−0.0072
TM	0.0229	0.4084	1.6429	−0.0029	−0.0084
KT	0.0255	0.4131	2.8526	−0.0055	−0.0131
ANA	0.0271	0.4127	3.4300	−0.0071	−0.0127
r-FR	0.0480	0.4226	12.4679	−0.0280	−0.0226

**Table 13:** Reactivity ratios obtained in the imposed conditions of LC2.

Method	*r*_1_	*r*_2_	*F^c^* × 1000	Bias	
				*r*_1_	*r*_2_
e-KT	0.7196	0.9203	0.0650	0.0004	−0.0003
k-NN	0.7243	0.9209	0.3595	−0.0043	−0.0009
TM	0.7268	0.9225	0.5268	−0.0068	−0.0025
ANA	0.7271	0.9227	0.5431	−0.0071	−0.0027
KT	0.7273	0.9229	0.5583	−0.0073	−0.0029
FR	0.7278	0.9237	0.5806	−0.0078	−0.0037
r-FR	0.7287	0.9235	0.8035	−0.0087	−0.0035
EVM-CN	0.7101	0.9187	0.9031	0.0099	0.0013

**Table 14:** Reactivity ratios obtained in the imposed conditions of LC3.

Method	*r*_1_	*r*_2_	*F^c^* × 1000	Bias	
				*r*_1_	*r*_2_
e-KT	0.6446	2.1078	0.3420	0.0054	0.0122
k-NN	0.6420	2.0820	0.9015	0.0080	0.0380
r-FR	0.6462	2.0564	2.3640	0.0039	0.0636
ANA	0.6566	2.0697	2.5141	−0.0066	0.0503
KT	0.6590	2.0699	2.7393	−0.0090	0.0501
TM	0.6710	2.1014	2.8394	−0.0210	0.0186
FR	0.6656	2.0811	2.9866	−0.0156	0.0389
EVM-CN	0.6087	2.2652	9.3730	0.0413	−0.1452

**Table 15:** Reactivity ratios obtained in the imposed conditions of MC1.

Method	*r*_1_	*r*_2_	*F^c^* × 1000	Bias	
				*r*_1_	*r*_2_
e-KT	0.0196	0.4023	0.3956	0.0004	−0.0023
EVM-CN	0.0215	0.4095	1.1558	−0.0015	−0.0095
FR	0.0310	0.4152	4.1969	−0.0110	−0.0152
k-NN	0.0314	0.3976	4.6885	−0.0114	0.0024
TM	0.0324	0.4202	4.8062	−0.0124	−0.0202
ANA	0.0470	0.4378	9.8920	−0.0270	−0.0378
KT	0.0513	0.4473	11.3844	−0.0313	−0.0473
r-FR	0.0813	0.4608	22.7203	−0.0613	−0.0608

**Table 16:** Reactivity ratios obtained in the imposed conditions of MC2.

Method	*r*_1_	*r*_2_	*F^c^* × 1000	Bias	
				*r*_1_	*r*_2_
k-NN	0.7258	0.9254	0.3599	−0.0058	−0.0054
e-KT	0.7136	0.9181	0.4728	0.0064	0.0019
EVM-CN	0.7231	0.9298	0.5635	−0.0031	−0.0098
TM	0.7438	0.9289	1.6691	−0.0238	−0.0089
ANA	0.7446	0.9286	1.7487	−0.0246	−0.0086
KT	0.7451	0.9287	1.7898	−0.0251	−0.0086
r-FR	0.7434	0.9278	2.0845	−0.0234	−0.0078
FR	0.7523	0.9372	2.0870	−0.0323	−0.0172

**Table 17:** Reactivity ratios obtained in the imposed conditions of MC3.

Method	*r*_1_	*r*_2_	*F^c^* × 1000	Bias	
				*r*_1_	*r*_2_
EVM-CN	0.6513	2.1301	0.2756	−0.0013	−0.0101
e-KT	0.6356	2.0962	0.8539	0.0145	0.0238
k-NN	0.6601	2.0404	3.7553	−0.0101	0.0796
FR	0.6869	2.0146	7.0124	−0.0369	0.1054
KT	0.6930	2.0244	7.1906	−0.0430	0.0956
ANA	0.6971	2.0279	7.4231	−0.0471	0.0921
TM	0.6804	1.9862	7.4980	−0.0304	0.1338
r-FR	0.6927	2.0254	7.7087	−0.0427	0.0946

**Table 18:** Reactivity ratios obtained in the imposed conditions of HC1.

Method	*r*_1_	*r*_2_	*F^c^* × 1000	Bias	
				*r*_1_	*r*_2_
k-NN	0.0407	0.3881	10.4452	−0.0207	0.0119
FR	0.0567	0.4630	15.9725	−0.0367	−0.0630
TM	0.0578	0.4676	16.3916	−0.0378	−0.0676
KT	0.0722	0.4858	21.0164	−0.0522	−0.0858
ANA	0.0772	0.4811	22.3837	−0.0572	−0.0811
r-FR	0.1602	0.5277	47.3857	−0.1402	−0.1277
e-KT	0.0001	0.0001	146.3598	0.0199	0.3999
EVM-CN	0.0001	0.0001	146.3598	0.0199	0.3999

**Table 19:** Reactivity ratios obtained in the imposed conditions of HC2.

Method	*r*_1_	*r*_2_	*F^c^* × 1000	Bias	
				*r*_1_	*r*_2_
e-KT	0.6973	0.9152	1.5737	0.0227	0.0048
EVM-CN	0.6925	0.9037	1.6354	0.0275	0.0163
k-NN	0.7650	0.9300	3.0243	−0.0450	−0.0100
FR	0.7644	0.9262	3.1204	−0.0444	−0.0062
KT	0.7732	0.9361	3.4138	−0.0532	−0.0161
ANA	0.7732	0.9350	3.4487	−0.0532	−0.0150
TM	0.7721	0.9320	3.4720	−0.0521	−0.0120
r-FR	0.7794	0.9396	4.4174	−0.0594	−0.0196

**Table 20:** Reactivity ratios obtained in the imposed conditions of HC3.

Method	*r*_1_	*r*_2_	*F^c^* × 1000	Bias	
				*r*_1_	*r*_2_
EVM-CN	0.6045	2.1281	4.0967	0.0455	−0.0081
e-KT	0.5701	2.0704	5.7943	0.0799	0.0496
k-NN	0.7284	2.0565	7.9745	−0.0784	0.0635
TM	0.7202	1.7780	16.3783	−0.0702	0.3420
ANA	0.7101	1.7543	16.5100	−0.0601	0.3657
KT	0.7123	1.7556	16.6245	−0.0623	0.3645
FR	0.7268	1.7792	16.8110	−0.0768	0.3408
r-FR	0.7170	1.7584	17.3594	−0.0670	0.3616

**Table 21:** The reactivity ratios obtained for copolymerization of NPEA-NVP [39].

Method	*r*_1_	*r*_2_	*F^c^* × 1000	Reference
k-NN	0.7892	1.0818	11.9489	this work
TM	0.8021	1.0844	11.9945	this work
ANA	0.7560	1.0205	12.3116	this work
e-KT	0.7420	1.0101	12.4438	this work
FR	0.7500	0.9900	12.8969	[39]
r-FR	0.6874	0.9484	14.0570	this work
KT	0.7200	0.9400	14.0804	[39]
EVM-CN	0.8919	1.0104	19.0656	this work

**Table 22:** The reactivity ratios obtained for copolymerization of Is-GMA [40].

Method	*r*_1_	*r*_2_	*F^c^* × 1000	Reference
k-NN	0.1130	0.2228	17.2372	this work
KT	0.1210	0.2240	17.5698	[40]
TM	0.1190	0.2480	17.9278	[40]
FR	0.1150	0.2060	17.9350	[40]
e-KT	0.1240	0.1980	19.2480	[40]
ANA	0.1468	0.2272	20.4971	this work
r-FR	0.2380	0.3160	36.7301	[40]
EVM-CN	0.0001	0.0001	102.9142	this work

**Table 23:** The reactivity ratios obtained for copolymerization of NIPAM-NVP [41].

Method	*r*_1_	*r*_2_	*F^c^* × 1000	Reference
e-KT	0.8380	1.1050	2.8946	[41]
k-NN	0.8618	1.0754	3.8764	this work
EVM-CN	0.8608	1.1899	5.0659	this work
TM	0.8862	1.0726	5.3121	this work
r-FR	0.8563	1.0314	5.6978	this work
ANA	0.8837	1.0610	5.7219	this work
KT	0.8888	1.0613	6.0057	this work
FR	0.9227	1.1055	6.0544	this work

## Data Availability

Not applicable.
